# Medical Opinion and Movement

**Published:** 1907-03-16

**Authors:** 


### Medical Opinion and Movement.

A question of considerable public importance has been taken in hand by the *Tribune*, and a meeting of influential members both of the medical and political worlds was held at the offices of the paper to consider and discuss means of preventing the admission of young children to public-houses. In the poor districts of London and other large cities women, accompanied by their small offspring, invade the public-houses and dose the children as well as themselves with gin or other forms of alcoholic poison. A resolution was proposed and carried unanimously that no child under fourteen years of age should be allowed into any licensed house other than a residential hotel. Among those present were Sir Thomas Barlow, Sir Lauder Brunton, Sir Victor Horsley, Mr. Franklin Thomasson, M.P., Mr. Macnamara, M.P., the Bishop of Hereford, and Professor Sims Woodhead. The conference was formed into a committee, and it is to be hoped that legislation may be effected in the sense of the resolution.

M. P. Emile Weil claims to have discovered a powerful hæmostatic in normal blood serum. It is already known that blood that has lost its power to coagulate can be made to clot *in vitro* by the addition of fresh serum. M. Emil Weil has found that the addition of fresh normal serum to blood from hæmophilics also increases *in vitro* its power of coagulability. From this observation he proceeded to treat hæmophilics and hæmorrhagic conditions by injections of blood serum, with results quite in accordance with his expectations. In his paper read before the Société Médical des Hôpitaux of Paris he gives several cases illustrative of the success of the treatment. By previous injections of blood serum hæmophilics have been able to undergo operations, such as tooth extraction, opening of empyema and incision of a perinephritic abscess without any excess of hæmorrhage. Cases of purpura, hæmaturia, and other hæmorrhagic conditions following fevers have yielded to the treatment. Human serum is found to be the most efficacious, but rabbit and horse serum may also be used. Ox serum is not to be recommended, as it produced certain ill-effects. For adults fifteen cubic centimetres may be introduced intravenously, or thirty subcutaneously, at a time. In children half-doses should be given.

The Erasmus Wilson Lecture on Pyorrhœa Alveolaris, recently delivered by Mr. Kenneth W. Goadby before the Royal College of Surgeons, will serve again to call the attention of the profession to the importance of this disease, and the influence it may exert, even in its mildest forms, on the general health of the individual. The profession has been somewhat slow to realise the far-reaching effects of any chronic local suppurative condition, especially of the mouth, on the general system. Mr. Goadby has been a pioneer in this respect in his combined capacity as a dental surgeon and a bacteriologist. He has made for years past a special study of the bacteria of the mouth, and his researches on these lines have placed him in a position of authority to speak on the subject. These researches have not led to the identification of any particular micro-organism with the disease, but suggest that the disease may be set up by a variety of bacteria, varying in different cases. By the use of the opsonic index Mr. Goadby has been able to arrange these different organisms in groups. What is, however, of special interest is the practical bearing of this work on the treatment of the disease.

The author points out that while in some cases local treatment—extraction, curetting, electrolysis, and local applications—may succeed in cutting short the disease, especially when applied in the early stages, in many the disease continues to progress, and the general symptoms of anæmia, neurasthenia, and chronic rheumatism persist in spite of these measures. These facts the author ascribes to the lowered resistance of the individual to the infective organism. His method of treatment is to isolate the infecting organisms of the patient and estimate the patient's opsonic index to the different groups, and then prepare a vaccine corresponding to the organism to which the patient's blood shows the lowest opsonic index. This vaccine is then injected, and the injections are controlled in the usual way by estimating variations in the opsonic index of the patient's blood during the treatment. The author cites a few cases of remarkable recovery from chronic septic conditions by this treatment. The treatment is, of course, laborious and requires considerable technical skill. But one has only to consider the deplorable condition of such cases of chronic septic poisoning, often resulting in permanent disablement, and reflect on the probability that a certain proportion at least of them may originate in some such local infection, to realise the importance of an efficient method of treatment, such as Mr. Goadby appears to herald forth.

According to recent researches of MM. Calmette and Guerin, tubercular infection of the bronchial glands, and consequently of the lungs, may take place *via* the mesenteric glands by absorption from the intestines without leaving ocular evidence of any lesion along the path of infection. Calves and young goats were given tubercle bacilli with their food, and as a result the tracheo-bronchial glands became enlarged, but there was no apparent lesion of the mesenteric glands. By inoculation, however, of these glands into guinea-pigs, tuberculosis was produced. Similarly inoculation of the mesenteric glands of children suffering from tracheo-bronchial adenopathy produced the same result. Since experiments have not definitely proved contagion by the respiratory tract, the authors suggest that it is more probable that children and adults contract the disease by swallowing infected material, whether food or milk of tubercular origin, or particles of human tubercular sputum. By similar experiments with pneumococci, M. Calmette is led to conclude that pneumonia and other acute affections of the lungs may be caused by infection through the same channels.

